# Upper Gastrointestinal Endoscopy Detection of Synchronous Multiple Primary Cancers in Esophagus and Stomach: Single Center Experience from China

**DOI:** 10.1155/2012/432367

**Published:** 2012-03-27

**Authors:** Rui Wang, Mo-Jin Wang, Jin-Lin Yang, Cheng-Wei Tang

**Affiliations:** ^1^Department of Gastroenterology and Endoscopy Center, West China Hospital, Sichuan University, Chengdu 610041, China; ^2^Department of Gastrointestinal Surgery, West China Hospital, Sichuan University, Chengdu 610041, China

## Abstract

The present study was undertaken to clarify the prevalence and clinicopathological features of synchronous multiple primary cancers (SMPCs) under upper gastrointestinal endoscopic examination. We enrolled 45,032 consecutive patients who underwent upper gastrointestinal endoscopic examination for digestive disease from January 2006 to December 2007 in our hospital and analyzed the clinicopathological features of SMPCs in esophagus and stomach. SMPCs are defined as two or over two different cancerous lesions developing in the same or other organs within 6 months. SMPCs were identified in 46 patients (0.1%). The gender ratio was 5.6 : 1 (male/female) and the mean age was 59.4 years. Synchronous esophageal and gastric cancers were the most frequent, being seen in 32 patients (0.07%). The most common histological types of SMPCs were squamous cell carcinoma in esophagus and adenocarcinoma in stomach, respectively. There were 27 (59%) SMPCs patients who had the history of simultaneous exposure to tobacco smoking and alcohol drinking. Additionally, 32 (78%) esophageal squamous cell cancers were associated with tobacco use. And 23 adenocarcinomas of the stomach were associated with *Helicobacter pylori *infection.

## 1. Introduction

In China, gastric cancer remains the first cause of mortality of cancer-related death. Meanwhile the incidence of esophageal cancer which happens in China accounts for the highest morbidity worldwide [1]. With the advance in upper gastrointestinal endoscopic technology combined with the detailed pathological examinations of biopsy specimens, the synchronous multiple primary cancers (SMPCs) in esophagus and stomach therefore have been increasingly detected, resulting in an increase in the incidence.

According to the criterion of Warren and Gates [2], the SMPCs are defined as two or over two different cancerous lesions developing in the same or other organs synchronously. Although the number of reports of MPCs in esophagus and stomach is growing [3–6], very few described the prevalence and clinicopathological features of SMPCs under upper gastrointestinal endoscopic examination, especially from China—high-risk region for both esophageal and stomach cancers. We, herein, reported a case series using a database that included a total of 45,032 consecutive patients who underwent upper gastrointestinal endoscopic examination for upper gastrointestinal diseases and analyzed the clinicopathological features of SMPCs in esophagus and stomach.

## 2. Material and Methods

A total of 45,032 consecutive patients who underwent upper gastrointestinal endoscopic examination for digestive disease from January 2006 to December 2007 at the Endoscopy Center of West China Hospital, Sichuan University (Chengdu) were enrolled in this study.

Endoscopic examination of the esophagus, stomach, and duodenum was performed with video endoscopes (Lucera, CV-260SL, Olympus, Japan) by experienced endoscopists. According to Japanese classification, esophageal cancers were divided into four types depending on the lesion's macroscopic appearance: fungating, ulcerating, infiltrating, and polypoid. Endoscopic gross pathologic presentations of early gastric cancers are classified according to the criteria proposed by Japanese Society for Gastroenterology as type I (protruded), type II (including IIa superficial elevated, IIb superficial flat, type IIc superficial depressed), and type III (excavated). For the advanced gastric cancer, the Borrmann classification was applied to describe the gross appearance. Borrmann type I represents polypoid or fungating lesions; type II is ulcerating lesions surrounded by elevated borders; type III represents ulcerating lesions with infiltration into the gastric wall; type IV is diffusely infiltrating lesions; type V is lesions that do not fit into any of the other categories.

Biopsy specimens were taken from all focal lesions. One specimen from the antrum was used for determination of *Helicobacter pylori* (HP) status by the rapid urease test. The biopsy specimens were fixed in 10% buffered formalin or 95% ethanol, embedded in paraffin, cut in 5-mm sections, and stained with hematoxylin and eosin. All specimens were evaluated by a single pathologist who was blinded to the clinical characteristics of the patients. All the patients were interviewed to obtain information on smoking status, drinking status, and past medical history by questionnaires. As a regular follow-up program for patients with esophagus cancer or gastric cancer, a combination of blood chemistry test, chest X-ray, upper gastrointestinal endoscopy, and thoracic/abdominal CT was performed.

Multiple primary cancers were defined according to the criteria of Warren and Gates: (1) all cancers must be malignant as determined by histologic evaluation; (2) each cancer must be geographically separate and distinct, and the lesions should be separated by normal-appearing mucosa; (3) metastasis cancer must be differentiated from multiple primary cancers and ruled out. SMPCs were defined as these cancers diagnosed within 6 months.

## 3. Results

### 3.1. Characteristics of SMPCs

Of the 45,032 patients who underwent upper gastrointestinal endoscopic examination, SMPCs were detected in 46 patients (0.1%). In the 46 patients with SMPCs, 39 patients were males, 7 were females (gender ratio of 5.6 : 1), and the average age of the patients with MPC was 59.4 (range 45–76 years).

### 3.2. Sites of SMPCs

The sites of SMPCs are summarized in Table 1. In our study, synchronous esophageal and gastric cancers were the most frequent, being seen in 32 patients (0.07%), including 7 in esophagus and cardia, 1 in esophagus and fundus, 11 in esophagus and body, 6 in esophagus and stomach angle, and 7 in esophagus and pylorus. We also found that 14 patients had the double primary esophageal cancer.

### 3.3. Endoscopic Gross Pathologic Presentations of SMPCs

In total, we found 60 lesions in esophagus which could be classified in terms of gastroscopic gross pathologic presentations: 24 fungating lesions, 16 ulcerating lesions, 19 infiltrating lesions, and 1 polypoid lesion. There were 10 cases of early gastric cancers (4 II c lesions and 6 III lesions) and 22 cases of advanced gastric cancers (7 Borrmann I lesions, 8 Borrmann II lesions and 7 Borrmann III lesions).

### 3.4. Histology of SMPCs

The histologic findings revealed 53 cases of squamous cell carcinomas, 6 of adenocarcinomas and 1 of undifferentiated in esophagus; 25 of adenocarcinomas, 4 of squamous cell carcinomas, 1 of adenosquamous cell carcinoma, 1 of undifferentiated, and 1 of signet ring cell carcinoma in stomach (Table 2). These lesions were localized at cardia (squamous cell carcinoma, *n* = 3), fundus (squamous cell carcinoma, *n* = 1), and body (adenosquamous, *n* = 1). The synchronous cancer of cardia carcinomas were located at upper (*n* = 1) and middle (*n* = 2) esophagus and were squamous cell carcinomas. The synchronous cancer of fundus cancer was located at middle esophagus and was also squamous cell carcinoma. The synchronous cancer of gastric adenosquamous carcinoma was located at middle esophagus and was adenocarcinoma. There were 11 SMPCs patients (11/14) with the same histology (squamous cell carcinoma) and 3 patients with different histology (squamous cell carcinoma and adenocarcinoma) in double primary esophageal cancer. In addition, there were 7 SMPCs (7/32) with the same histology in different organs: 4 of squamous cell carcinomas and 3 of adenocarcinomas.

### 3.5. Tobacco Smoking, Alcohol Drinking, and Hp Infection in SMPCs

We found 35 ever-smokers (76%) and 32 ever-drinkers (70%) in patients with SMPCs. The number of cigarettes taken by ever-smokers per day was 18.6 ± 6.5. The daily ethanol intake was 56.6 ± 41.7 g. There were 27 (59%) SMPCs patients who had the history of simultaneous exposure to tobacco smoking and alcohol drinking. Additionally, 32 (78%) esophageal squamous cell cancers were associated with tobacco use. We detected 38 (83%) SMPCs patients with HP infection. In these cases, 23 adenocarcinomas of the stomach were associated with HP infection.

## 4. Discussion

Ever since Billroth, quoted by Boice et al. [7], reported the existence of double successive cancers in the same patient in 1889, the MPCs in esophagus and stomach have become no longer rare. Yoshino et al. [3], in the first research of MPCs patients with gastric cancer, reported that the gastrointestinal tract, including esophageal (3rd place), is the most common site of multiple combined with gastric cancer. In another study, 4 of 2,509 gastric cancer patients (incidence 0.2%) were found to have primary esophageal cancers [4]. For the esophageal cancer, gastric cancer is the most common second primary cancer according to a series of reports. The incidence of esophageal cancer that coincides with gastric cancer, reported by several authors, varies between 1.4% and 5.2%. In the present study, we found 32 patients with SMPCs in esophagus and stomach (0.07%, 32/45032). There are two reasons for relatively low relevance ratio in our study: (1) the previous investigation population was patients with esophageal or stomach cancer, on the other hand we collected patients who underwent upper gastrointestinal endoscopic examination; (2) we did not enroll the patients with metachronous MPCs.

The patients with SMPCs in esophagus and stomach were found more often to be male and elderly. These results were consistent with those of previous studies [4–6]. In this paper, the gender ratio was 5.6 : 1 (male/female) and the mean age was 59.4 years in the patients with SMPCs of esophagus and stomach. Although little is known of the definite causes of SMPCs, a number of studies have described SMPCs as the result of interaction between genetic factors of the host together with dietary and other risk factors in the environment. We detected 38 (83%) SMPCs patients with HP infection. In these cases, 23 adenocarcinomas of the stomach were associated with HP infection. However, this result must be interpreted with caution, because the prevalence rate of HP infection may be underestimated by the rapid urease test. Our results showed that 59% SMPCs patients had the history of simultaneous exposure to tobacco smoking and alcohol drinking. Recently, reviews of epidemiological evidence lent strong support to the effects of smoking and alcohol consumption in the development of MPCs [8]. This view is supported by Wen et al. [9] who pointed out that Northern China, the formerly known esophageal cancer epidemic region, was actually a high-risk region for both esophageal and stomach cancers. These similar geographic distributions suggested that the environmental risk factors, including tobacco and alcohol, have the common carcinogenetic effect to the whole upper gastrointestinal tract.

Through the observation of endoscopic presentations combined with surgical specimens, we found esophageal lesions were more severe and more extensive than stomach. Patients, who usually had solid food dysphagia which appeared earlier than stomach symptoms, visited the hospital and received the endoscopic examination. These situations may result in increased possibility of missing the lesions beneath esophagus.

## 5. Conclusions

SMPCs in esophagus and stomach were found in 0.1% of the patients who underwent upper gastrointestinal endoscopic examination. The most common histological types of SMPCs were squamous cell carcinoma in esophagus and adenocarcinoma in stomach, respectively.

##  Conflict of Interests 

The authors declare that there is no conflict of interests.

## Figures and Tables

**Table 1:** Sites of SMPCs in patients who underwent endoscopic examination.

Site 1	Site 2			
	Esophagus			
	Upper	Middle	Low	Total
Stomach				32
Cardia	2	3	2	7
Fundus	0	1	0	1
Body	1	6	4	11
Angle	0	2	4	6
Pylorus	0	4	3	7
Esophagus				
Upper	0	1	2	3
Middle	0	1	3	4
Low	1	4	2	7

Total	4	22	20	92

**Table 2:** Histopathologic diagnosis of SMPCs in esophagus and stomach.

Histological type	Site	
	Esophagus	Stomach
Adenocarcinoma	6	25
Squamous cell carcinoma	53	4
Adenosquamous cell carcinoma	0	1
Signet ring cell	0	1
Undifferentiated	1	1
